# Development and optimization of oleogel made with soy protein isolate and xanthan gum using emulsion template approach and its comparison with solid fats

**DOI:** 10.1016/j.heliyon.2024.e25224

**Published:** 2024-01-29

**Authors:** Saumya Sonam Sinha, Ashutosh Upadhyay, Anurag Singh

**Affiliations:** a Department of Food Science and Technology, National Institute of Food Technology Entrepreneurship and Management, Kundli, Sonipat (Haryana), India; b Department of Food Technology, Harcourt Butler Technical University, Nawabganj, Kanpur (Uttar Pradesh), India

**Keywords:** Oleogel, Saturated fats, Rheology, Microstructure, Texture, Unsaturated fats

## Abstract

This study aims to develop oleogel as a potential substitute for solid fats in the diet. A novel combination of unmodified Soy Protein Isolate (SPI) and Xanthan Gum (XG) have been utilized to gelate sunflower oil, using an emulsion template approach. The experimental trials employing Response Surface Methodology are conducted to optimize various parameters that affect the oil binding capacity, textural and rheological properties of the oleogel. The concentration of soy protein varies in the range of 5–15 %, the ratio of soy protein to xanthan gum ranges from 1:2 to 1:4, and the ionic strength varies from 0.2 to 1 M. The goal is to formulate oleogel that closely resembles solid fats. Responses namely the oil binding capacity and gel strength value of oleogel were observed best fitted to a linear model whereas, the hardness of oleogel found following a quadratic model. The SPI-XG combination was found effective in entraping more than 95 % of the oil. The best formulation of SPI: XG, 1:4; SPI concentration, 15 % and ionic strength of 1.0 M with 95.5 % of oil retention and hardness and gel strength value comparable to commercial solid fats.

## 1 Introduction

In the present world, health problems like cardiovascular disease, diabetes and obesity are associated with the intake of quantity and quality of fat. Numerous studies have revealed that the intake of saturated fats and trans fats must be limited for good cardiac health [1]. Solid fats such as butter, *ghee/butteroil*, and shortenings are rich in saturated fats in contrast to edible oils. They are widely acknowledged for their relative oxidative stability, desirable texture, spreadability, fair shelf life, and distinct flavour profile. This sets them apart from liquid oils, which may cause oil leakage and weak textural properties in the final product [2]. On the other hand, liquid vegetable oils contain a substantial amount of polyunsaturated fatty acids (PUFA), which have been shown to be effective in combating cardiovascular disease and cancer [3]. As a balance approach, in order to achieve the desired functional and textural properties resembling solid fats, researchers have shown their interest in the creation of solid fats with added health benefits, aiming to boost the consumption of beneficial fats.

Different methods have been developed to turn liquid vegetable oils into solid fats. One of such method is oleogelation which is getting the attention of scientists as a potential alternative of saturated and *trans*-fat [2]. Oleogels are simply gels of edible oils or referred to as a semi-solid lipophilic composition formed by encapsulating liquid oil within the three-dimensional (3D) framework established by a gelator or a combination of gelators known as oleogelators [[4], [5], [6]]. Solid/semisolid structure from liquid oils is achieved using oleogelators. These substances have the ability to gelatinize or thicken oils, transforming them into gel-like structures. Several oleogelators have been tried for oleogelation. Oleogelators are classified as Low Molecular Weight Oleogelators (LMWO), organic compounds (molecular weight <3000 Da), and High Molecular Weight Oleogelators (HMWO) compounds, also known as polymeric compounds. The LMWO organizes themselves in oil to form a fixed crystalline network. The interactions reported in LMWO supported oleogels are hydrogen bonds, hydrophobic bonds, and van der Waals forces, etc., under control temperature. Examples of LMWO include monoacylglycerols, fatty acids, fatty alcohols, natural waxes, phytosterols and oryzanol [7,8]. The HMWO includes polymers like proteins, polysaccharides, and binary and ternary combinations of these. Although HMWO provide good visco-elastic properties but as they are hydrophilic, they are difficult to incorporate into the oil. So, different methods like emulsion template, foam template, and solvent exchange methods are used for developing oleogels [[9], [10], [11]].

The complex of protein and polysaccharide as an oleogelator has been well documented in the literature [[12], [13], [14]]. Present study explores a novel combination of soy protein isolate and xanthan gum as an oleogelator for PUFA rich sunflower oil. Soy protein isolate used in this study is a plant-based protein and has been used for developing oleogel considering its amphiphilic nature and usage as an emulsifier in food. The ability of soy proteins to emulsify can be enhanced through chemical, physical, and enzymatic modifications. Addition of polysaccharides is one of the ways to enhance the emulsifying activity and stability of the emulsion [15]. Hence, xanthan gum can function in this complex as a thickening agent. Improved aqueous phase's viscosity maintains the physical stability of emulsion over the long run by reducing the movement of droplets [16]. Recent studies have shown the ability of XG and protein conjugates to promote oil structure [8,10,12]. Thus XG-protein complexes are likely to facilitate oleogel formation and could be explored.

Numerous research findings have substantiated that the characteristics of emulsions formed by protein-polysaccharide complexes are notably influenced by both pH levels and ionic strength [17]. According to the findings, SPI-xanthan gels made without salt were mostly maintained by non-covalent (H-bonding and hydrophobic) and SS bond interactions, whereas gels made with salt require electrostatic interactions to keep the gel structure stable. The portion that was likely electrostatically connected to the xanthan was the -7S subunit [18]. Hence, only pH 3 is used in all formulas chosen for the present investigation. The effect of ionic strength was also investigated in this study. As per the best of our knowledge, oleogel formation using this protein and polysaccharide combination in the unmodified form and the effect of ionic strength on oleogel formation has not been described in the literature.

In this study, the objective was to create an oleogel using sunflower oil with the intention of mimicking the characteristics of a solid fat. A combination of soy protein isolate and xanthan gum was employed as oleogelators, and the emulsion template method was utilized. On the basis of preliminary trials in emulsion, pH was fixed as 3. The selected pH might favouring electrostatic attraction between SPI and XG as XG is an anionic polysaccharide and SPI is cationic at pH 3. To optimize the ratio, protein concentration, and ionic strength of the oleogelators, response surface methodology was employed. The resulting optimized oleogel was also compared with commercially available solid fats in the market, facilitating an assessment of its similarity to solid fats and paving the way for potential applications in food products.

## 2 Materials and methods

### 2.1 Materials

‘Nutrivita’ brand soy protein isolate (SPI, 91 % protein) was purchased online from Amazon India. Xanthan gum (Viscosity: ≥1200.00 cP) was purchased from Hi- Media Pvt. Ltd. (India). Sunflower oil, butter, margarine, and shortening were procured from the local markets. All chemicals used in the study are of analytical grade. Distilled water was used to prepare all dispersions.

### 2.2 Methods

#### 2.2.1 Preparation of dispersion and emulsion

Stock solution (15 % concentration) of SPI was made by adding the desired amount of SPI in distilled water and the stirring of the solution was continued for 1 h at room temperature (22 °C). Xanthan gum was gradually dissolved in hot water (80 °C), while agitated under a magnetic stirrer to make the XG solution of the desired concentration (0.25 %). Afterward, these solutions were kept at 4 °C for overnight to ensure complete hydration. Emulsions were prepared by ‘layer by layer’ technique [10] where sunflower oil (φoil = 0.12) was first added in aqueous solution of SPI then XG solution was added into it and homogenized (Ultraturrax, IKA T18, Germany) it at 11,000 rpm for 10 min at room temperature (22 °C). Afterward, the desired pH (3) of the solution was maintained by adding HCl (0.1 M) to it. Ionic strength was maintained by NaCl solution of the desired molars.

#### 2.2.2 Optimization of oleogel

A three-level Box-Behnken design with three numerical factors— A-polysaccharide to protein ratio (XG: SPI, 2:1–4:1) B- protein concentration (SPI, 5–15 %), and C- ionic strength (0.2–1.0) was used to study the impact of independent factors on the production of oleogel (Table 1). The range of each parameter was selected on the basis of preliminary experiments conducted. There were 17 runs in the matrix design. The experimental design was carried out using Design-Expert Software 13.0. Response Surface Methodology (RSM) to optimize the process conditions for the preparation of the emulsion design parameters. The experiments occurred at random.

**Table 1:** Coded values and actual values for selected independent parameters.

Coded value	Actual Value		
	A	B	C
+1	4	15	1.0
0	3	10	0.6
−1	2	5	0.2

#### 2.2.3 Oleogel preparation

The emulsion was then oven dried at 70 °C [10] until a stable sample weight was achieved. A glass rod was then used to shear the dried sample, releasing the oleogel. For further analysis, all developed oleogels were stored at 4 °C.

### 2.3 Analysis of response parameters

#### 2.3.1 Oil binding capacity (OBC, %)

The oil binding capacities of oleogels were calculated by centrifugation method [19] using the following formula:

(1) $$\text{OBC}=100-\frac{\left[\left(b-a\right)-\left(c-a\right)\right]}{\left(b-a\right)}\times 100$$

Where a = weight (g) of empty centrifuge tubes, b = weight (g) of tubes after putting the sample in them, and c = weight (g) of tubes after the centrifugation process and removing extra oil from them.

#### 2.3.2 Hardness

Texture analyzer (Texture Exponent v.6.1.4.0, Stable Microsystems) with a cylindrical probe (P/6), connected to a 5 kg load cell, was used to conduct a penetration test in order to examine the textural characteristics of the oleogels and commercial solid fats. Trigger force of 1 g and a speed of 1 mm/s, the penetration distance set for the experiment was of 10 mm. The highest force recorded was regarded as the firmness (g) [19].

#### 2.3.3 Gel strength

Using a Modular Compact Rheometer (Anton Paar, Rheoplus software 3.21), with an application temperature unit integrated with a Peltier system, rheological tests of the samples were carried out. All experiments were completed at 25 ± 0.01 °C. Storage modulus was used to determine the gel strength. Amplitude and frequency sweeps were used to compare the rheological properties of optimized oleogel and commercial solid fats. Amplitude sweep tests were accomplished at 0.01–100 % strain range by keeping the frequency constant at 1Hz and frequency sweep tests were performed at a frequency range of 0.1–100 Hz by keeping the strain constant at 0.1 % [13].

#### 2.3.4 Microstructure analysis

The microstructure of the optimized oleogel and emulsion were examined using confocal microscopy. A hydrophobic dye, Nile red (0.01 % w/v), was used to stain the oil. The dye was initially dissolved in sunflower oil to prepare the emulsion and dried sample. A Leica TCS SP8 confocal microscope available at Indian Institute of Technology- Delhi (IIT-D), Sonipat, was used to image the samples. Ar laser (543 nm) was used for the excitation process and the confocal images were obtained at 60 × magnification for each sample [10].

#### 2.3.5 Statistical analysis and model validation

Experimental data were analyzed using Design Expert software using RSM were presumed to be represented as under:

(2) $$R=\beta_{0}+\beta_{1}A+\beta_{2}B+\beta_{3}C+\beta_{12}\text{AB}+\beta_{13}\text{AC}+\beta_{23}\;\text{BC}+\beta_{11}\;A^{2}+\beta_{22}\;B^{2}+\beta_{33}\;C^{2}$$

Where *R* is representing the response parameters which are a function of the independent parameters (*A,B,C*) that have been taken as independent factors [20], *β*_0_ (intercept) is the regression coefficient of each equation which consists of linear, interaction, and quadratic terms represented as *β*_1_, *β*_2_, *β*_3_, *β*_12_, *β*_13_, *β*_23_, *β*_11_, *β*_22_, *β*_33_ whereas Analysis of variance (ANOVA) was used to assess the accuracy and significance of the fitting models. To assess significance, R Squared (R2), adjusted- R2, predicted- R2, lack of fit value (LOF), and adequate precision (AP) were used. The suggested model and LOF should, respectively, be significant (p value ≤ 0.05) and insignificant. The difference between predicted- R2 and adjusted- R2 shouldn't be greater than 0.2 and AP value greater than 4 is preferable [21].

Furthermore, the same software was used to optimize the parameters. In order to validate the model, three experiments were again performed using the optimized parameters. The percentage error between the predicted and experimental values was determined to see whether the model was acceptable (see Table 5).

## 3 Results and discussion

### 3.1 Optimization of oleogel

Effect of process variables on oleogel properties is shown in Table 2. The experiments were conducted as per the experiment design described in Section 2.2.2. All the independent variables are represented in coded form. Each experimental observation was recorded in triplicate and an average value is reported in Table 2.

**Table 2:** The RSM design for the optimization of oleogel indicates independent variables and the selected response.

Independent Experimental Variable			Responses (R)		
A	**B**	**C**	**R1** **OBC (%)**	**R2** **Hardness (g)**	**R3** **Gel strength (Pa)**
+1	0	+1	92.3 ± 1.2	250.907 ± 35	129800 ± 500
0	0	0	84.4 ± 2.3	54.906 ± 13	108800 ± 421
0	+1	−1	90.4 ± 0.9	306.878 ± 62	110110 ± 389
0	0	0	82.2 ± 3.1	63.906 ± 25	108800 ± 415
0	+1	0	82.06 ± 0.9	41.913 ± 36	108300 ± 330
+1	+1	0	98.5 ± 2.5	185 ± 52	148000 ± 287
−1	+1	0	83.1 ± 3.9	91.23 ± 12	176100 ± 463
+1	−1	0	65.6 ± 1.3	43.631 ± 16	629.6 ± 104
0	0	0	86.4 ± 2.1	59.06 ± 20	108800 ± 398
0	+1	+1	87.33 ± 1.7	26.79 ± 33	211600 ± 265
−1	0	−1	83.4 ± 1.3	269.33 ± 41	91830 ± 154
+1	0	0	82.69 ± 3.3	105.946 ± 25	121000 ± 254
−1	−1	0	58 ± 0.4	20.32 ± 11	768 ± 112
0	0	0	83.5 ± 2.8	52.85 ± 11	108800 ± 400
0	0	0	80.4 ± 2.2	56.79 ± 15	108800 ± 392
0	−1	−1	55.4 ± 0.9	45.59 ± 16	5876 ± 201
−1	0	+1	77.7 ± 1.8	100 ± 21	108900 ± 366

In order to arrive at a predictive model for each of the responses, the data was statistically analyzed and presented in Table 3. Each of the responses is being discussed further individually.

**Table 3:** Coefficients of the model (in terms of coded factors) and statistical coefficients for each response variable.

	OBC			Firmness			Gel Strength		
Source	CE	SS	SE	CE	SS	SE	CE	SS	SE
Intercept	+79.60*	1483.15	–	+49.93*	1.305E+05	–	+9561.67*	1.422E+10	–
A	+4.38*	150.24	2.39	+26.19*	4579.57	6.98	−973.30**	7.417E+06	9029.56
B	+12.67**	1218.89	2.43	+42.68*	9756.58	7.79	+6574.13*	3.283E+10	9168.31
C	+1.85*	19.60	2.79	−43.23*	6271.10	9.85	+2961.03*	5.041E+09	10540.17
AB	–	–		+17.61*	1241.12	9.02	**-**		
AC	–			+45.61**	4297.17	12.55	**-**		
BC	–			−92.65**	16357.14	13.07	**-**		
A [2]	–			+56.77**	9769.09	10.36	**-**		
B [2]	–			−27.87*	2318.05	10.44	**-**		
C [2]	–			+107.30**	38409.28	9.88	**-**		
R-Squared	0.7177			0.9828			0.8372		
Adj R-Squared	0.6525			0.9608			0.7996		
Predicted R-Squared	0.4782			0.7636			0.7029		
Adequate Precision	10.4930			20.2639			15.5627		

Note.

* Significance level at (*p* < 0.05).

** Significance level at (*p* < 0.1).

Abbreviations: CE, coefficient estimate; SE, standard error; SS, sum of squares.

#### 3.1.1 Oil binding capacity

**Fig. 1:**
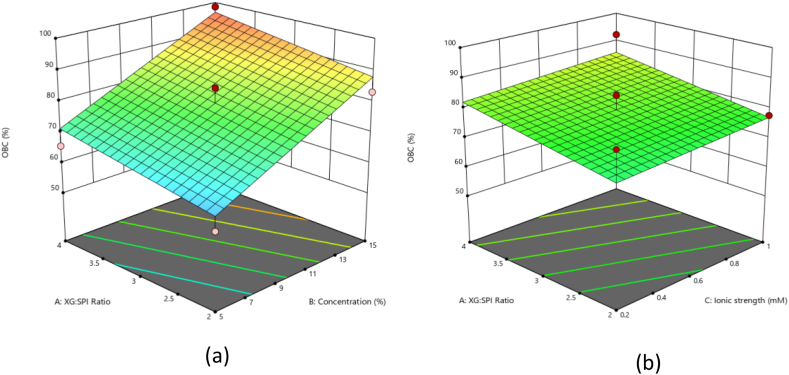
Response surface plot showing (a) the effect of SPI concentration, and ratio of XG:SPI on OBC at constant concentration of ionic strength (0.6) (b) the effect of XG:SPI and ionic strength on OBC of oleogel at constant concentration of SPI (10 %).

OBC is the most important characteristic of oleogel, the higher the value of OBC, better the gel would be on account of holding oil as a solid fat option. OBC values in this study were found to be in the range of 55.4–98.5 % (Table 2). Statistical analysis suggested a better fit of the linear model (P-value <0.05 and coefficient of determination (R^2^) of 0.7177) to predict OBC. The difference between the predicted R^2^ of 0.4782 is in reasonable agreement with the Adjusted R^2^ of 0.6525; i.e., the difference is less than 0.2. Adequate Precision (AP) measures the signal-to-noise ratio where the value greater than 4 is desirable. AP ratio of 10.493 indicates an adequate signal. The OBC value was minimum when XG:SPI ratio, SPI concentration and ionic strength were 3, 5 % and 0.2 M, respectively, whereas OBC was maximum when XG:SPI ratio was 4, SPI concentration was 15 % and ionic strength was 0.6 M (Table 2). To visualize the effects of variations in process variables on OBC, response surfaces were generated (Fig. 1a), it can be seen that both the concentration of SPI and XG:SPI have a positive effect on the oil binding capacity of oleogel i.e., increasing their values resulting in the increase in the OBC values however the concentration has more positive effect in comparison to the XG:SPI ratio, the possible reason for this could be more the protein concentration more is the availability of hydrophobic groups that bind to oil [22] and also due to higher amount of protein accumulation at oil droplet interface [23]. The addition of polysaccharides has found to increase the OBC as found in studies by Yu et al., 2021, where they found out that combining soy protein with different polysaccharides can increase the OBC values to more than 97 %. Alizadeh et al. [24], have also stated the same findings that increasing sodium caseinate (protein) and HPMC (polysaccharide) concentration has a synergistic effect which results in more oil binding up to 97 %. The polysaccharide interacts with the protein and makes the interface more stable, also the unadsorbed polysaccharides get filled in between the droplets of emulsion which creates a steric hindrance that results in less coalescence between droplets and hence more oil binding [14,25].

The effect of ionic strength on the oleogel formation as can be seen from the response surface graph (Fig. 1b) is also having a positive effect as at 0.2 M the OBC value is 83.4 %, at 0.6 M it is 84.4 % and at 1 M of NaCl the OBC value is 92.3 %. The control oleogel prepared without any salt also confirms that the salt has a positive effect on OBC, as the OBC value of the control sample was only 58 %. However, many studies have proved that large ionic strength causes the suppression of the protein –polysaccharide complex [26] because of the shielding of charged polymers by the salt, but ionic strength has also been shown to increase the hydrophobicity of SPI [27] which accounts for its more OBC value.

#### 3.1.2 Hardness

**Fig. 2:**
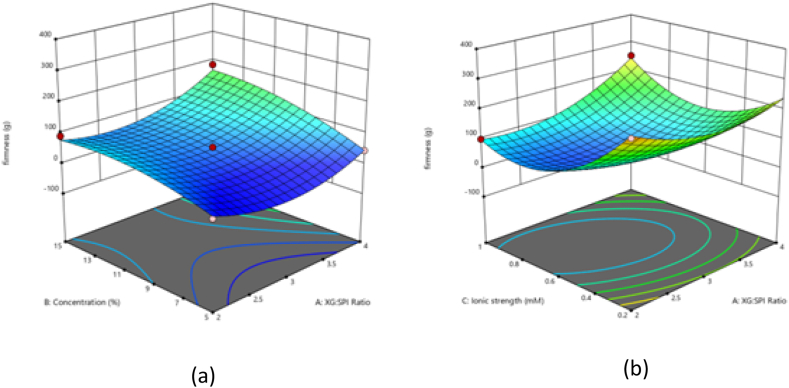
Response surface plot showing (a) the effect of SPI concentration and ratio of XG:SPI on firmness of oleogel at constant concentration of ionic strength (0.6) (b) the effect of XG:SPI ratio and ionic effect on the firmness of the oleogel at constant concentration of SPI (10 %).

Force of resistance to penetration is another significant textural characteristic to identify hardness of solid fats. Experimental oleogels had hardness values between 20.32 and 306.878 g at 25 °C (Table 2) while that of the control sample (without salt) was 100.54 g. The obtained data were fitted with a quadratic model and the predicted R^2^ of 0.7636 is in reasonable agreement with the adjusted R^2^ of 0.9608; AP ratio of 20.264 indicates an adequate signal (Table 3). The Model F-value of 44.55 implies the model is significant. There is only a 0.01 % chance that an F-value this large could occur due to noise. The lowest value of firmness of oleogel was when XG:SPI ratio, SPI concentration, and ionic strength were 2, 5, and 0.6, respectively, and the highest was when SPI:XG ratio, SPI concentration and ionic strength were 3,15 and 0.2 respectively. Fig. 2 (a) indicates that at a constant ionic strength of 0.6, it can be seen that the concentration of SPI has a more positive effect on the firmness value, then the ratio of polysaccharide and protein. Many authors have reported the same finding that the addition of polysaccharides increases the hardness of the oleogel [14,25,28]. Some of the relevant findings are reported as follows. The oleogel formulated with electrolyzed SPI exhibited a hardness value of 133.45 g. However, when various anionic polysaccharides were introduced, the hardness significantly increased to more than 300, as documented by Yu et al., 2021. Addition of citrus pectin, in an another study, at a concentration of 1.5 % resulted in a hardness value of 138.46 g, increasing the citrus pectin concentration to 4.5 % led to a substantial increase in hardness, reaching up to 320.48 g^25^. Fig. 2 (b) indicates the inverse effect of ionic strength on firmness of oleogel while SPI concentration was kept constant at 10 %, this could be attributed to the failure of protein-polysaccharide complexation.

#### 3.1.3 Gel strength

**Fig. 3:**
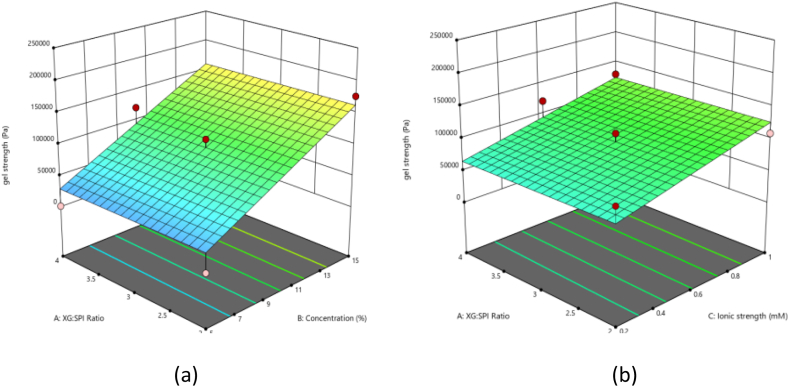
Response surface plot showing (a) the effect of SPI concentration and ratio of XG:SPI on gel strength of oleogel at constant concentration of ionic strength (0.6) (b) the effect of XG:SPI ratio and ionic strength on the gel strength of oleogel at constant concentration of SPI (10 %).

Dynamic rheological measurements provide useful data on the structural strength of the samples. The difference between G′ and G′′, the average value of G′ in the LVR (G′LVR), and the length of the linear part of the curve can all be used to determine the gel strength of a sample within this LVR [29]. The strain sweep test determines the storage modulus (gel strength) of oleogels in LVR. G’ values of the prepared oleogels were in the range of 629.6–211,600 Pa as listed in Table 2. Linear model was chosen as the best fit in this analysis with the coefficient of determination (R^2^) of 0.83. The Predicted R^2^ of 0.7029 is in reasonable agreement with the Adjusted R^2^ of 0.7996. AP ratio of 15.563 indicates an adequate signal. The highest value of gel strength was seen when XG: SPI ratio, SPI concentration, and ionic strength were 3, 15 %, and 1 M, respectively, and the lowest when XG: SPI ratio, SPI concentration, and ionic strength were 4, 5 %, and 0.6 M, respectively. From Fig. 3 (a & b), it can be seen that XG:SPI ratio has not that profound effect on the gel strength, whereas increasing SPI concentration results in increasing the gel strength of oleogel. Oleogels formulated with a combination of gelatin and flaxseed gum by a researcher also demonstrated a comparable storage modulus of around 20,000 Pa, using gelatin alone [28]. The addition of polysaccharides not always enhance the gel strength of the oleogel. A negative relationship was also observed in oleogels based on SPI and k-carrageenan, the G' value for SPI alone was approximately 100,000 Pa whereas, the combination of SPI and k-carrageenan yielded a G' value of around 6000 Pa^30^. However, the increase in protein concentration was always found to increase the gel strength of the oloegels. Study carried out on whey protein isolate and low methoxyl pectin found that an increase in total polymer concentration increased the storage modulus of the HIPEs however the study was not focused on the ratio of the respective polymers [30]. Another study by same group of authors stated the same finding as increasing sodium caseinate concentration lead to the development of firmer visco-elastic matrices holding the liquid oil well [23]. The ionic strength was found to have the positive impact on the gel strength of oleogel as high ionic strength was also found to increase the OBC, hence overall it was found to contribute to the formation of the stable interface between matrix and oil. However, the literature on the effect of ionic strength on emulsion based oleogel is very limited but it is clear that ionic strength greatly affects the electrostatic interaction between the polymers [31].

### 3.2 Multiresponse optimization

The goal of this optimization study was to forecast the ideal protein concentration and polysaccharide-to-protein ratio for creating oleogels that mimicked the rheological and textural characteristics of commercial fat. Hardness & gel strength should be chosen from a range and the OBC should be at its maximum. A desirability function above 0.77 was obtained in 71 solutions by numerical optimization. A point with maximum desirability of 1 was selected under the following setting: XG: SPI Ratio = 4, SPI concentration = 15 %, and ionic strength = 1. To ensure the validity of the predictive models, the oleogel sample was made in triplicate in accordance with this ideal environment. The findings of the experiment were given in Table 4. To ascertain whether there was a substantial discrepancy between these values and those anticipated by the model, a *t*-test statistical analysis was carried out. This test was unable to reveal any appreciable differences. The OBC of the sample prepared at optimization conditions was 95.5 %, which is in good harmony with the predicted value of 95.8 % (see Table 5).

**Table 4:** Validation of predicted values of responses obtained at the optimal level.

Optimized oleogel				
	**Observed parameters**	**Predicted values**	**Experimental values**	**Percent error**
Response parameters	**OBC (%)**	**95.802**	**95.5 ± 0.5**	**−0.31**
	**Firmness**	**75**	**78.4** ± **5.2**	**+4.5**
	**Gel Strength**	**184161.447**	**182400 ± 500**	**−0.95**

**Table 5:** Comparison of hardness of oleogel with other commercial solid fats.

Product	Hardness (g)	Adhesiveness	Springiness
Butter	72.13 ± 0.37^b^	133 ± 0.87^c^	0.45 ± 0.03^b^
Margarine	54.08 ± 0.40^c^	201.14 ± 0.79^b^	0.70 ± 0.03^a^
Oleogel	77.72 ± 0.63^b^	3.40 ± 0.06^d^	0.12 ± 0.01^c^
Shortening	250.41 ± 0.82^a^	301.29 ± 0.34^a^	0.15 ± 0.02^c^

Each value is indicated as mean ± SD; Significance level at (*p* < 0.05).

### 3.3 Evaluation of the optimized samples

#### 3.3.1 Microstructure and visual appearance of the obtained oleogel

**Fig. 4:**
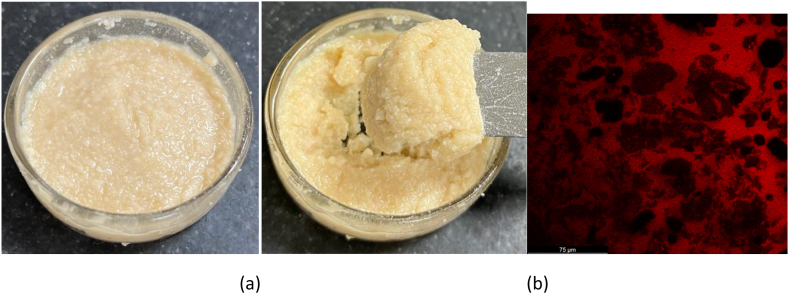
(a & b) Visual and confocal image of optimized oleogel.

Visual image of optimized oleogel is shown in Fig. 4a. Typically, when an oil-water emulsion is dried, the coalescence of oil droplets causes the macrophase of liquid oil to separate [10]. However, the incorporation of proteins in the oleogel as prepared in the study were able to hold oil (Fig. 4b), this could be attributed to the availability of the hydrophobic site of the protein and enhanced stability by XG-structuring agent. Hence, it was possible to create an acceptable oleogel from the designed emulsions. Sunflower oil that was liquid was successfully transformed into oleogel that resembled solids.

#### 3.3.2 Hardness

The purpose of this study is to develop oleogel that can replace commercial solid fats, so different types of commercial solid fats like shortening, margarine and butter were also compared with the optimized oleogel obtained from this study. The hardness of fats are mainly affected by the temperature of the measurement source and solid fat content [32]. The operational temperature, i.e., 25 °C was used to measure the hardness of the samples. When examining optimized oleogel alongside widely-used solid fats such as butter, margarine, and shortening, it was observed that its hardness, a crucial textural attribute for solid fats identification, closely resembled that of butter and margarine. However, shortening exhibited a notably higher hardness value in comparison. However, the adhesiveness and springiness of oleogel are not comparable to butter and margarine.

#### 3.3.3 Rheological properties

**Fig. 5:**
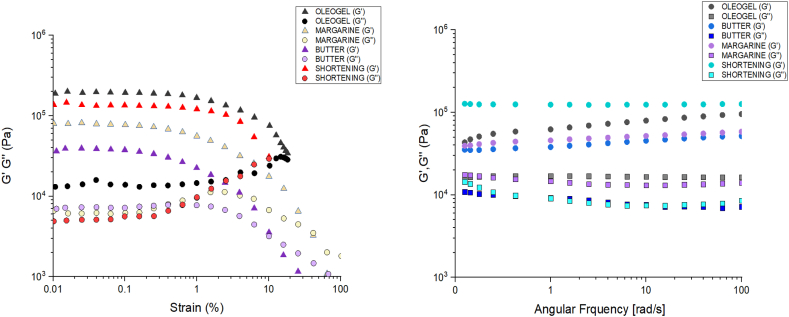
Data from **(a)** Strain sweep test **(b)** Frequency sweeps carried out on oleogel and other solid fats.

Fats are known to behave as stiff solids up until a deforming stress surpasses the yield value, at which time the product begins to flow like a viscous liquid. This flexibility results from the crystallised substance forming a network of fat crystals that retains liquid oil [33]. The strain sweeps test was used to determine the elastic modulus of solid fat and oleogel samples in the linear viscoelastic range (G’ LVR). Fig. 5a displays the amplitude performed over a strain range of 0.01 Pa–100 Pa, at 1 Hz of frequency, and 25 °C to identify the linear viscoelastic region. Two distinct regions were observed: (I) a linear viscoelastic region where the storage modulus (G') and loss modulus (G") were nearly constant, and (II) a nonlinear region where G' and G" started to decrease. G' was higher than G" in all samples in the linear viscoelastic range, which can be used as a measurement of gel strength and predominant elastic behavior. From Fig. 5 (a&b), it can be deducted that oleogel exhibits a predominant elastic behavior and higher gel strength in comparison to the other solid fats. As the strain increased, G′ values remained constant and decreased at high strain, indicating that the structure of the oleogel and commercial fats broke at high strain rate. Strong gels have G′ being greater than G′′ throughout the whole frequency range and an inversely proportional relationship between complex viscosity (η*) and rising angular frequency (ω). Further evidence that the oleogel samples do not exhibit a gel-to-sol transformation even at a higher rate of deformation comes from the absence of a cross-over point (G′ = G′′) even at high frequency values [34,35].

## 4 Conclusions

Oleogel made with combination of unmodified soy protein isolate and xanthan gum was successfully optimized using the Response Surface Methodology. The effects of SPI concentration, XG: SPI ratio and ionic strength on the oil binding capacity, gel strength, and hardness of oleogels were examined in this work using RSM. Additionally, the oleogel developed is enriched with a considerable amount of soy protein isolate, making it a commendable protein source alongside its provision of providing PUFA rich fat. Furthermore, the optimized oleogel were compared with the commercial solid fats to check the potential of optimized oleogel to replace commercial solid fats. According to the findings, it is possible to produce oleogel via the emulsion template method as a *trans*-free and low-saturated solid fat with the hardness similar to commercial butter available and visco-elasticity better than all the solid fats. This indicates its potential use as an alternative to solid fat. The sole limitation of this study was the grainy appearance of the oleogel, attributable to the high concentration of the protein. To overcome this challenge, potential solution could involve incorporating further modifications in gels/formation of gels, employing novel technologies like ultrasound treatment or high-pressure homogenization applied to the protein. These techniques are designed to expose more hydrophobic sites on the protein and reduce particle size. By doing so, not only can the concentration of the protein oleogelator be decreased, but the grainy appearance of the oleogel can also be mitigated.

## Data Availability Statement

The data will be made available on request.

## CRediT authorship contribution statement

**Saumya Sonam Sinha:** Data curation, Formal analysis, Investigation, Methodology, Writing – original draft. **Ashutosh Upadhyay:** Conceptualization, Project administration, Supervision, Validation, Writing – review & editing. **Anurag Singh:** Conceptualization, Supervision, Validation, Visualization.

## Declaration of competing interest

The authors declare that they have no known competing financial interests or personal relationships that could have appeared to influence the work reported in this paper.
